# The symbolic consumption processes associated with ‘low-calorie’ and ‘low-sugar’ alcohol products and Australian women

**DOI:** 10.1093/heapro/daad184

**Published:** 2023-12-30

**Authors:** Hannah Pitt, Simone McCarthy, Danica Keric, Grace Arnot, Sarah Marko, Florentine Martino, Julia Stafford, Samantha Thomas

**Affiliations:** Faculty of Health, Institute for Health Transformation, Deakin University, 1 Gheringhap Street, Geelong, Victoria, 3220, Australia; Faculty of Health, Institute for Health Transformation, Deakin University, 1 Gheringhap Street, Geelong, Victoria, 3220, Australia; Cancer Council Western Australia, Level 1/420 Bagot Road, Subiaco, Western Australia, 6008, Australia; Faculty of Health, Institute for Health Transformation, Deakin University, 1 Gheringhap Street, Geelong, Victoria, 3220, Australia; Faculty of Health, Institute for Health Transformation, Deakin University, 1 Gheringhap Street, Geelong, Victoria, 3220, Australia; Faculty of Health, GLOBE, Institute for Health Transformation, Deakin University, 1 Gheringhap Street, Geelong, Victoria, 3220, Australia; Cancer Council Western Australia, Level 1/420 Bagot Road, Subiaco, Western Australia, 6008, Australia; Faculty of Health, Institute for Health Transformation, Deakin University, 1 Gheringhap Street, Geelong, Victoria, 3220, Australia

**Keywords:** alcohol, women, marketing, commercial determinants of health

## Abstract

The influence of commercial actors, practices and processes on the health and wellbeing of women is still not well understood. The alcohol industry has developed a range of products to appeal to new ‘health conscious’ markets, such as ‘low-calorie’ and ‘low-sugar’ products. While these products may have specific appeal for women, there has been little in-depth research that has sought to understand how women conceptualize these products and the range of symbolic meanings that women associate with these products. An online qualitatively led survey was conducted with *n* = 497 Australian women who had consumed alcohol in the last year. Questions related to the reasons for and influences on alcohol use, the purchasing of ‘low-calorie’ or ‘low-sugar’ products and the influence that these products might have on women’s alcohol use. Data were interpreted using reflexive thematic analysis. Women consumed alcohol to relax, cope with everyday stressors and because of the alignment with social practices and social connection. Women perceived that these products provided a healthier alternative to traditional alcohol products and that they aligned with women’s values relating to weight and the thin ideal. Some women were concerned that these products could increase alcohol consumption by reducing the perceptions of risk associated with alcohol. Policy consideration is needed to address how product claims and attributes may influence population groups’ interpretations of the risks and benefits of these alcohol products, including the illusion that these products have protective potential and are better for overall health and wellbeing.

**Contribution to Health Promotion**

- This research contributed to an understanding of how commercial actors may align their products with the health beliefs and aspirations of women.
- Alcohol products marketed as ‘low-calorie’ or ‘low-sugar’ may appeal to women particularly in relation to health goals relating to weight, and social values associated with the thin ideal.
- There is some evidence that women perceive that these products may reduce perceptions about the broader risks associated with alcohol use and may contribute to the increased consumption of alcohol.
- Consideration is needed about the impact of promoted product attributes on women’s alcohol beliefs and consumption patterns.

## BACKGROUND

There is growing global recognition of the impact that commercial actors have in shaping health and equity (Gilmore *et al.*, 2023). While research has documented the negative impacts of harmful industries on health, limited attention has been given to the unique implications of corporate practices on population subgroups—including women (McCarthy *et al*., 2023). Despite women facing increased risks of health and social inequities, the extent to which commercial actors contribute to these risks remains inadequately understood (Hill and Friel, 2020; McCarthy *et al.*, 2023). Public health researchers have documented the range of tactics that harmful industries have used to both target women and shape social norms associated with their products (McCarthy *et al*., 2023). The alcohol industry provides a clear recent example of how commercial actors may strategically target and influence women’s alcohol consumption behaviours through the development of novel products and promotions (Atkinson *et al*., 2022; Cao *et al.*, 2023; Hill and Friel, 2020).

Women are increasingly targeted by the alcohol industry as valuable consumers of their products (Atkinson *et al*., 2022; Cancer Council WA *et al*., 2019; Emslie *et al.*, 2012). There is now compelling evidence that the gender gap associated with alcohol use is closing (Slade *et al*., 2016; White, 2020), and they are drinking alcohol at higher rates than women from previous generations (White, 2020). There are a range of alcohol-related risk factors that are specific to women (Erol and Karpyak, 2015; Slade *et al*., 2016), including breast cancer (McCaul *et al*., 2019) and the risks associated with alcohol consumption during pregnancy, such as fetal alcohol spectrum disorder (Dejong *et al*., 2019; Heenan *et al*., 2022; Oei, 2020). Studies have demonstrated that a range of different population subgroups of women are drinking at levels that may pose significant risks to their health and wellbeing (Keyes *et al*., 2019). Particular concern has been raised about increases in alcohol consumption among middle-aged and older women (Kersey *et al*., 2022; Lunnay *et al*., 2022; Miller *et al*., 2022) who have been largely overlooked in public health policy responses to alcohol product harms (Kersey *et al*., 2022; Nicholson *et al.*, 2017).

Women’s alcohol behaviours are influenced by a range of diverse social circumstances, environments and experiences (Kersey *et al*., 2023; Lunnay *et al*., 2023), which interact with commercial (or industry) practices, as well as government policy responses (Atkinson *et al*., 2019; McCambridge *et al.*, 2018). This interplay of social, environmental, commercial and political determinants can influence the decisions that women make about the risks and benefits of consuming alcohol (Dumbili, 2022; Kersey *et al.,* 2022; Lunnay *et al.*, 2022). For example, research clearly shows that alcohol use is seen as a valuable social connector for different groups of women (Kersey *et al.*, 2022; Wright *et al.*, 2022) and is used to foster group belonging and bonding (Nicholls, 2022) and facilitates a range of distinct social practices and identities, including what is considered socially acceptable drinking attitudes and behaviours (Armstrong *et al*., 2014; Dare *et al*., 2020; Dresler and Anderson, 2017; Lennox *et al*., 2018). For example, Lunnay and colleagues (2022, 2023) show that social class and life transitions contribute to the diverse reasons for drinking alcohol among those in middle life, including the role that alcohol plays in managing different life stages.

Social practices also intersect with the commercial tactics of the alcohol industry, with a range of novel products and promotions developed to symbolically respond to valued aspects of women’s identities (Hunt and Antin, 2019; Kersey *et al.*, 2022). Marketing theorists argue that these processes of ‘symbolic consumption’ occur when industries use marketing to influence and rework the social acceptance and cultural meanings attached to various products and brands (McCracken, 1986; Otnes and Scott, 1996). Consumers then use the social information that is attached to these products or brands to shape or align with their own values, beliefs and self-image (McCracken, 1986; Otnes and Scott, 1996). Therefore, the significance of consuming a certain product or brand may go beyond the basic functional value of the products to play an important role in identity construction (Fournier, 1998). These symbolic processes have been used by a range of industries, including tobacco (Kozinets *et al.*, 2019; Tokle and Pedersen, 2019), gambling (Deans *et al*., 2016; Pitt *et al.*, 2018) and the gun lobby (Yamane *et al*., 2020) to link health-harming products with a range of valued social attributes, including social status, group belonging, social worth, glamour and social acceptance (Gendall *et al.*, 2012; McCarthy *et al*., 2023).

Researchers argue that alcohol consumption for women goes beyond the functional consumption of the product and is closely linked to the construction of social identities (Emslie *et al*., 2015; Harding *et al.*, 2021; Kersey *et al.*, 2022; Lennox *et al*., 2018). Alcohol identities symbolically intersect with the marketing strategies and symbolic meanings that are developed by the alcohol industry to specifically appeal to different groups of women (Atkinson *et al.*, 2019; Bosma *et al*., 2022; Maani Hessari *et al.*, 2019; Nguyen, 2021). For example, alcohol has been promoted as an enhancer of social interactions and bonding among female friends, and positioned by marketers as a way for women to escape traditional gender roles (Atkinson *et al*., 2022). Companies have also aligned their brand marketing with feminine themes in an attempt to normalize and ritualize the use of their alcohol products (European Centre for Monitoring Alcohol Marketing, 2008; Ramšak, 2022). This has also been seen with the alcohol industry’s use of corporate social responsibility tactics that have aligned brands with women’s causes and events (Atkinson *et al.*, 2022; Petticrew *et al.*, 2018), including International Women’s Day and cancer charities (Cousins, 2022; Mart and Giesbrecht, 2015).

Symbolic messages about alcohol are not just given through advertising and promotions, but also in the products that are developed. For example, flavoured alcohol beverages including ready-to-drink (RTD) or pre-mixed alcohol products have a unique appeal for young women, including the use of sugary flavours and captivating product designs (Atkinson *et al.*, 2019; Brain *et al.*, 2000; Mosher and Johnsson, 2005). Certain product packaging such as ‘slim’ alcohol cans are more appealing to girls than boys (Purves *et al*., 2014). ‘Pink drinks’ have value for young women because of their ‘instagrammability’ (Cancer Council WA *et al*., 2019), and also appeal to women embracing both empowered and feminine identities (Atkinson *et al*., 2022).

Most recently, alcohol products have been developed to respond to changing consumer preferences relating to overall health and wellbeing (Keric and Stafford, 2019). These ‘better-for-you’ products are promoted in ways that draw upon health or nutrient claims or are associated with sport, exercise and healthy lifestyles (Nicholls, 2022). We define ‘better-for-you’ alcohol products as:

Alcohol products that are: (i) marketed as having a range of health-related attributes, including those with low sugar, carbohydrate or calorie content, the use of natural or organic ingredients, the lack of additives or preservatives, or that are broadly associated with better health and wellbeing; and (ii) are developed and marketed in a way that creates the illusion of a healthier product.

In Australia, nutrient information panels are not mandatory on alcohol products. While health claims are not permitted, nutrient claims relating to calories, carbohydrates or gluten can be used on products with 1.15% and above alcohol content (FSANZ, 2022). Due to concerns relating to health and nutrient claims, there are changes proposed to the Food Standards Code that include exploring the need for more specifications relating to carbohydrate and sugar claims, and energy labelling on alcohol products (FSANZ, 2022). Some researchers have suggested that the development of ‘low-calorie’ and ‘low-sugar’ products is a deliberate health-washing strategy employed by the alcohol industry to give an ‘illusion of healthfulness’ without addressing the alcohol content of the products (Haynes *et al*., 2022; Keric *et al*., 2022). Some researchers argue that the marketing focus on nutritional attributes (such as claims on low-carbohydrate/low-sugar content) is problematic because it creates a perception that some types of alcohol products can be consumed without (or with minimal) health risks (Keric and Stafford, 2019). In doing so, these products may ultimately contribute to the increased consumption of alcohol (Shemilt *et al*., 2017). Some researchers have also shown that nutrition content claims can lead to misperceptions about the alcohol content within a product. For example, Cao and colleagues (2023) found that young women who viewed alcohol products with low-sugar claims thought that these products would assist with weight management and were lower in alcohol content than regular alcohol products. They also rated these products as being healthier and/or less harmful to health. The authors concluded that the marketing of these products could exploit the motivations of consumers who wanted to improve their health and manage their weight (Cao *et al*., 2023).

These studies provide important information about how the alcohol industry may adapt their products to respond to consumer values. The following study develops on these existing studies by qualitatively examining how women conceptualize ‘low-calorie’ and ‘low-sugar’ alcohol products, including whether they perceive that these products align with their broader health and social values, and whether these products may influence women’s alcohol attitudes and behaviours. Three research questions guided the study:

1. What do women describe as the main influences on their alcohol consumption?
2. How do women conceptualize the appeal of ‘low-calorie’ and ‘low-sugar’ alcohol products?
3. Is there evidence that there are processes of symbolic consumption occurring which align ‘low-calorie’ and ‘low-sugar’ alcohol products with women’s broader health and social values?

## METHODS

### Approach

The data presented in this article were part of a broader online qualitative panel survey that explored Australian women’s alcohol attitudes and behaviours, with a specific focus on alcohol products and marketing. The current article focuses on exploring women’s attitudes towards ‘low-calorie’ or ‘low-sugar’ alcohol products. These two attributes were selected as they have been the focus of other studies investigating the nutritional claims of these products (Keric *et al*., 2022) and quantitative investigations examining the impact of these products on women (e.g. Cao *et al*., 2023). In Australia, 'low-sugar' claims have been found to be on 5.9–6.9% of beer, cider and RTD, with around 20% of RTD including a sugar claim. Low-risk ethical approval was received from the Deakin University Human Ethics Advisory Group.

### Online qualitative surveys

An online qualitatively led survey was chosen for this exploratory study. These types of surveys, which are focused predominately on open-text questions, enable researchers to collect broad qualitative insights from a diverse range of people in a timely and cost-efficient manner (Braun *et al*., 2021). Qualitative surveys have been shown to be useful when exploring sensitive topics (McCarthy *et al*., 2022), and may engage people in qualitative research who otherwise would not volunteer to be part of focus groups or lengthy one-to-one interviews. The anonymous nature of these surveys means that participants are free to respond to questions honestly and without judgement, potentially reducing power imbalances that participants can feel when interacting directly with a researcher (Braun *et al*., 2017). While this method has some limitations, such as the lack of opportunity to ask follow-up questions, Braun *et al.*, (2021) highlight that these types of surveys have the ability to reach broad population groups, and provide rich qualitative insights.

### Sample and recruitment

Purposive sampling was used to recruit Australian women who had consumed alcohol in the last year. A sample size of *n* = 500 was the target for this study, which was considered sufficient to provide enough information power to answer the aims and research questions of the broader study (Malterud *et al*., 2016). To be included in this study, women were required to be aged 18 years or older (the legal age to purchase alcohol in Australia), be an Australian resident and have sufficient English language proficiency. Alcohol consumption in the last year was the screening criterion to ensure a broad range of alcohol experiences and attitudes were included in the sample. Soft quotas were used to ensure diversity of the sample by age group and geographical location (typical of state and territory populations).

Qualtrics, an online survey platform and panel company, was engaged to host the survey and recruit participants. Qualtrics shared a link to the survey with partner panel companies, who distributed this to potential participants who met the study inclusion criteria. Potential participants were able to download the Plain Language Statement and were required to click that they consented to participate in the study prior to beginning the survey. Consenting individuals were asked two screening questions about gender and alcohol use. Those who did not identify as female, or who had not consumed alcohol in the last year were screened out and were not able to complete the survey.

### Data collection

Data were collected between September and October 2022 (taking on average 26 min to complete). The survey was piloted with 61 participants in September 2022, to ensure it was technically sound and that open-text questions elicited the intended types of responses. Following a review of the pilot data, minor wording changes such as ‘*do you think*’ to ‘*how do you think*’ and ‘low-calorie’ or ‘low-sugar’ products might ‘*influence women’s alcohol use*’ rather than ‘*appeal to women*’, were made to ensure that they elicited more open and targeted responses. Pilot responses were then discarded before the full launch. The initial round of data collection led to *n* = 509 completions. The research team then reviewed these responses to ensure that all participants had made genuine attempts to complete the survey. A further 12 participants were subsequently removed due to incomplete responses or nonsensical data.

Participants were asked discrete questions relating to sociodemographic factors (age, state of residence, education, employment) and alcohol use (frequency, preferred alcohol product and purchase of ‘low-calorie’ or ‘low-sugar’ products). Open text questions related to the main reasons for drinking alcohol, reasons for purchasing (or not purchasing) ‘low-calorie’ or ‘low-sugar’ alcohol products and perceptions about the influence of these products specifically for women and their alcohol use. The broader survey included a range of pictorial examples of different alcohol marketing strategies. In relation to the content of this article, a social media post was shared which showed a RTD alcohol product containing claims of ‘*no sugar*’ and ‘*only 85 calories*’ written on the picture.

### Data analysis

SPSS was used to calculate basic descriptive statistics and frequencies from the quantitative data.

The dataset was downloaded to Microsoft Excel which was used to manage the qualitative analysis process. Braun and Clarke’s (2022) six steps of reflexive thematic analysis were used to guide the analysis and to construct themes in relation to the research questions. They also highlight the importance of researchers recognizing and acknowledging the theoretical assumptions that underpin the analysis; in this study, concepts from symbolic consumption theory were used to guide our coding and theme development (Braun and Clarke, 2022). A detailed description of the application of these steps is reported in Supplementary File 1.

## RESULTS

### General characteristics

Table 1 reports the sociodemographic and alcohol use characteristics of the sample. Responses from *n* = 497 women (18–88 years old, *M* = 46.1; SD: 17.67) were included in the final analysis. Most participants lived in Australia’s three largest states (New South Wales *n* = 157, 31.6%; Victoria *n* = 126, 25.4%; Queensland *n* = 106, 21.3%). Women’s alcohol use ranged from less than once a month (*n* = 90, 18.1%) to every day (*n* = 22, 4.4%), with half of the participants drinking alcohol at least once a week. Participants’ *most* preferred alcohol product was wine (*n* = 195, 39.2%), followed by spirits (*n* = 102, 20.5%). Just under half of women had ever purchased a ‘low-calorie’ or ‘low-sugar’ alcohol product (*n* = 228; 45.9%). Almost three-quarters of young women aged 18–34 years had purchased a ‘low-calorie’ or ‘low-sugar’ alcohol product (*n* = 109; 72.2%), compared to 41.7% of 35–54 years (*n* = 73) and 26.9% of 55 years and over (*n* = 46).

**Table 1:** : Sociodemographic and alcohol use characteristics

Demographic	Number	Percentage
**Age**		
18–34	151	30.4
35–54	175	35.2
55+	171	34.4
**State**		
NSW	157	31.6
VIC	126	25.4
QLD	106	21.3
WA	44	8.9
SA	38	7.6
ACT	12	2.4
TAS	12	2.4
NT	2	0.4
**Education**		
Year 12 or below	161	32.4
Certificate I, II, II, IV	108	21.7
Diploma/Advanced diploma	65	13.1
Bachelor’s degree	113	22.7
Grad diploma/Certificate	12	2.4
Postgraduate degree	38	7.6
**Employment**		
Working full-time	186	37.4
Working part-time/casually	124	24.9
Unemployed but looking	13	2.6
Homemaker	42	8.5
Retired	101	20.3
Full-time student	8	1.6
Other	13	2.6
**Alcohol frequency**		
Less than once a month	90	18.1
About 1 day a month	52	10.5
2–3 days a month	103	20.7
1–2 days a week	138	27.8
3–4 days a week	60	12.1
5–6 days a week	32	6.4
Everyday	22	4.4
**Alcohol product most preferred**		
Wine	195	39.2
Spirits	102	20.5
Pre-mixed spirits	72	14.5
Cocktails	44	8.9
Beer	43	8.7
Cider	41	8.2
**Purchased low calorie/low sugar**		
Yes	228	45.9
No	269	54.1

A summary of the key themes of the study, with illustrative quotes, is presented in Supplementary File 2.

### Reasons for alcohol use

To understand how women conceptualized their own alcohol use, including the reasons that they engaged with alcohol, three themes and smaller sub-themes were constructed from the data.

#### A mechanism for relaxing and coping with daily stressors

The main reason for consuming alcoholic products was to relax, with words such as ‘*relax*’, ‘*relaxing*’ and ‘*relaxation*’ mentioned multiple times by participants. Concepts associated with relaxing were linked to coping with stress, including a range of everyday pressures like work or parenting, and helping women unwind. For some, having a drink represented the end of a long workday or week and the beginning of leisure time. Some women stated that they used alcohol as a reward or treat for making it through a hard day at work or to help them relieve the stress of a long day. For example, *‘To relax after a long work week’.* For others, alcohol was used to cope with a range of health challenges including anxiety or chronic pain, such as the following participant who said:

*The main reason I drink alcohol is to relax, unwind and help with the pain I am in all the time*. – 50-year-old female

While some women stated that they only consumed alcohol on special occasions, such as a birthday or other special events, others stated that alcohol consumption was a normal and regular part of everyday activities such as watching sports, attending social activities, socializing with colleagues after work or cooking and eating a meal. The following older woman described the way alcohol was consumed alongside a social event marked by having a meal:

*As an accompaniment with a meal it can be most enjoyable, to share a glass with a friend over dinne*r. – 81-year-old female

#### Social practices, group connection and social rituals

For many women, alcohol was also associated with social practices, group connection and social rituals. Most perceived that social factors had the most significant impact on their drinking behaviours, including that the consumption of alcohol helped to facilitate social connections with friendship networks, family members or work colleagues—‘*To have a cold drink with friends on the weekend*’. Alcohol use was intertwined with increased social enjoyment and *‘to have fun’*, this was particularly reported for young women. This included acting as a disinhibitor which allowed them to ‘*let loose’* or *‘loosen up’.* Some reported that they used alcohol to build their self-esteem and confidence when attending social events, and as a tool to help reduce their anxiety in social situations. Alcohol was often described as enabling women to socialize—*‘to relax, gain confidence and be social’.* Other women commented on the peer pressure that they would feel to consume more alcohol than they normally would because of their husbands/partners or their friends. The following younger woman described drinking alcohol even when she was not intending to drink:

*If I go out with people and they all want a drink I would drink with them, even if I wasn’t overly keen on having a drink*. – 26-year-old female

While getting intoxicated was not an everyday occurrence for most women, some indicated that when they did consume alcohol their intention was to ‘*get drunk*’, feel intoxicated or to experience a *‘light buzz’.* For example, the following participants referred to getting ‘*drunk*’ in their answers:

*I love the taste of Sambuca and I love being drunk. –* 29-year-old female

*The main reason I drink alcohol is to get drunk. I don’t particularly love the taste and I don’t like to consume unnecessary calories so I only tend to drink if I’m looking to get drunk. –* 26-year-old female

*… occasionally I like to get blind drunk.* – 47-year-old female

#### Reasons for reducing alcohol consumption

Some women articulated a change in their alcohol behaviours due to a range of health and social issues. Parenting responsibilities and motherhood had a key influence on alcohol consumption. A few women stated that they wanted to be able to ‘*focus*’ on parenting and set a good example for their children. Other mostly older women reported that their alcohol consumption had reduced due to health concerns or conditions such as anxiety, arthritis and liver problems. A few women stated that they rarely drank because they had witnessed alcohol dependence or the negative behavioural consequences of drinking among their children, siblings, friends, or parents:

*I think that alcoholics in my family limit my alcohol intake because I am scared I will become like them. –* 22-year-old female

### Engagement with and perceptions of ‘low-calorie’ and ‘low-sugar’ alcohol products

To better understand women’s engagement with and perceptions of ‘low-calorie’ and ‘low-sugar’ alcohol products, three interlinking themes and sub-themes were constructed from the data.

#### An alternative for those looking for a ‘healthier’ option

Some women believed that ‘low-calorie’ or ‘low-sugar’ products provided an alternative for women who needed to change their alcohol consumption, or who were becoming more health or ‘*body conscious*’. For example, women stated that they had purchased ‘low-calorie’ or ‘low-sugar’ products because they were experiencing ‘*stomach issues*’ and bloating or health issues such as diabetes. There was a clear set of health-related values that linked the consumption of ‘low-calorie’ and ‘low-sugar’ alcohol products with a perception that women cared about their health or fitness. Participants thought that these products would be influential for women because they were *‘healthier’*, *‘better for you’*, *‘not as bad for you’*, and did not contain unhealthy ingredients or *‘bad stuff’*. While women across a range of ages discussed how these products aligned with health, younger women particularly emphasized the health benefits of ‘low-calorie’ and ‘low-sugar’ products.

*Women will buy it because its healthier*. – 65-year-old female

*More women will use these, the idea of them not being “unhealthy” means more likely to use them. –* 29-year-old female

*More appealing as not intaking more sugar and bad stuff.* – 19-year-old female

A few participants also considered the appeal of these products in relation to the effects of alcohol that they experienced the next day. These participants thought that these products could influence women because they could drink without the ‘*hangover*’ or reported purchasing these products because it meant they did not ‘*… feel as bloated or sick the next day*’. Some participants, particularly younger women, stated that drinking these types of products made them feel ‘*less guilty*’ about their alcohol consumption. This was either because they felt that they were consuming fewer calories or because they believed that the alcohol product was healthier for them. For example, participants perceived that ‘low-calorie’ or ‘low-sugar’ alcohol products would enable women to ‘*indulge*’ without the perceived negative consequences of the high sugar and calorie content of regular alcohol products:

*I wanted the same effects, but I didn’t want to feel bad about myself*. – 27-year-old female

However, a few women were sceptical about the true health benefits of these products. For example, this included that the sugar substitutes used in these products could negatively impact health or that they contained ‘*other bad ingredients to compensate*’. The following participant thought that ‘low-calorie’ or ‘low-sugar’ products could lead women to focus on the nutritional claims of the product rather than the alcohol content which was more harmful to health:

*I think they would only focus on the low cal and not the alcohol content.* – 60-year-old female

#### Symbolically aligning with values about body weight and the thin ideal

Most women thought that ‘low-calorie’ or ‘low-sugar’ alcohol products would influence women’s alcohol consumption because they could positively impact and facilitate weight loss and weight management. This perception was consistent across all age groups, regardless of whether they had previously purchased a ‘low-calorie’ or ‘low-sugar’ alcohol product. Women stated that these products had a particular benefit for women who were trying to watch ‘*their sugar intake*’ or ‘*keep calories down*’. For women who had purchased ‘low-calorie’ or ‘low-sugar’ products, the main reason for doing so was that they were on a diet, trying to lose weight or reduce their calorie consumption. There were many beliefs that these products benefited weight loss and maintenance. Some and particularly those who had purchased these products believed that the products could be used to help women achieve a thin body ideal, including helping them to ‘*stay skinny*’, and preventing weight gain:

*So I don’t get too fat*. – 58-year-old female

Some women perceived that these products would appeal to women because of the societal pressure for women to be concerned about their weight. Participants perceived that these products aligned with diet culture, and a focus on weight and body image. Some women reported that these products would be influential to women who had insecurities about their bodies, who wanted to ‘*look their best*’, and to those who valued being ‘*slim*’. There were exaggerated perceptions from some participants that women would find these products appealing because so many were concerned about sugar, calorie content, the amount that they consumed and weight loss. The following younger woman who had purchased a ‘low-calorie’ or ‘low-sugar’ product, highlights the pressure women can be under to reduce calories:

*Women are already taught to eat less and be very calorie focused because of society so women may be more likely to choose low calorie alcoholic products to reduce calorie intake*. – 27-year-old female

#### Normalizing drinking through increased social acceptance and a reduced perception of risk

Some participants were concerned that the ‘low-calorie’ or ‘low-sugar’ content of products could encourage women to drink more. This was commonly described among women in middle adulthood (35–54 years) compared to other age groups. Participants suggested that women might perceive that ‘low-calorie’ and ‘low-sugar’ products eliminate the need to worry about calories or weight gain. While participants referred to the influence these claims could have on other women, the focus on sugar content or calories was also reflected in the reasons why women had purchased these products. Additionally, participants also stated that women may overlook the risks associated with the alcohol content of these products due to the emphasis in messaging on ‘low-calorie’ or ‘low-sugar’ claims. Women thought that these labels or claims could create a perception that women could *‘feel better’* about drinking alcohol, and ultimately encourage women to consume more alcohol:

*Low in calories or sugar would perhaps make them consume more than they normally would due to the famous word LOW. –* 66-year-old female

*It suggests that drinking low/no sugar calorie alcohol is better therefore can be consumed in greater amounts. –* 52-year-old female

Some participants perceived that ‘low-calorie’ and ‘low-sugar’ alcohol products could also play a role in the increased social acceptance and normalization of alcohol. This perspective was more prevalent among middle-aged to older women. A few commented that the presence of these products reinforced social norms associated with having to drink to fit in or feel included in social situations especially when on a diet:

*I think it increases alcohol use, as that* [sugar/calorie content] *used to be a big reason why people wouldn’t drink. –* 28-year-old female

*It brings looking a certain way and having fun but not drinking the calories so can be drunk on any lifestyle.* – 27-year-old female

They stated that ‘low-calorie’ or ‘low-sugar’ drinks enabled women to ‘*let go*’, enjoy themselves and be ‘*drunk*’. Some women thought that women would consume these drinks to get intoxicated, or could drink more than what they normally would without any negative implications for their weight:

*Gets you fucked up without the calories*. – 33-year-old female

*So many drinks are high in sugar and calories, low cal or low sugar drinks would encourage women who are watching their weight to let go and drink a few more drinks. –* 21-year-old female

## DISCUSSION

This project aimed to provide qualitative insights about symbolic processes that influence women’s attitudes towards and consumption of ‘low-calorie’ and ‘low-sugar’ alcohol products. The study shows that there are clear intersections between the product attributes and claims of ‘better-for-you’ products, and the social factors, health values and the perceived benefits of consuming these products for women. While younger women were more likely to report purchasing these products, the overall sense of why ‘low-calorie’ and ‘low-sugar’ products would influence women’s alcohol consumption was similar across age groups. Supplementary File 3 provides a model demonstrating the symbolic processes associated with ‘low-calorie’ and ‘low-sugar’ alcohol products and women. The model visually depicts the way that alcohol marketing is using the attributes of the symbolic consumption theory (values, beliefs, self-image; cultural meanings; novel products and brands) in the design and marketing strategies for ‘better-for-you’ alcohol products. This includes how women’s perceptions of ‘better-for-you’ products, particularly for this study ‘low-calorie’ and ‘low-sugar’ alcohol products, provide an alternative for those looking for a healthier option, aligned with female body image and the thin ideal, and may be normalizing these products through increased social acceptance and reduced perceptions of risk. The way that women engage with these messages about ‘better-for-you’ products may be creating a health halo effect which can reduce perceptions of risk associated with this product and increase overall product use.

This study provides evidence that ‘low-calorie’ and ‘low-sugar’ alcohol products symbolically align with women’s health and social values. It provides evidence to suggest that the marketing tactics of the alcohol industry, including the development of novel products, may undercut health risk messages by aligning products that appeal to wellness and ‘take-care-of-yourself’ ideals. The article provides preliminary evidence to suggest that these industry tactics may be altering women’s attitudes about the risks associated with these products and the harms that they may cause. In doing so, the alcohol industry has provided a ‘solution’ to women’s desire to reduce the health risks associated with alcohol consumption, while ensuring that women continue to drink.

Similar to other studies, we found a ‘health halo’ effect associated with these products whereby women perceived them to be healthier, less risky or more beneficial to their wellbeing (and particularly their weight) than regular alcohol products (Cao *et al*., 2023; Keric *et al.*, 2022). Participants’ values associated with alcohol were highly gendered, with concerns relating to body image, appearance, preventing weight gain and using ‘low-calorie’ or ‘low-sugar’ alcohol products to feel less guilty about consuming products with high calories or sugar. These findings are somewhat unsurprising given research that has demonstrated that women are constantly exposed to messages which reinforce attitudes towards body image and the thin ideal (Aparicio-Martinez *et al*., 2019; Mills *et al*., 2017). These products were also perceived as a ‘healthier’ alternative and were viewed as preventing some of the negative aftereffects of alcohol such as hangovers or excess weight gain. Previous research has similarly found that alcohol products with low-sugar claims were perceived as being less harmful to health, healthier and more suitable for weight management (Cao *et al.*, 2023). Some women stated that the perception that these products were healthier, could lead women to consume more of the product. This suggests that these products may deflect the risks and harms associated with the alcohol content of the products. This finding supports previous concerns that ‘better-for-you’ claims may encourage an illusion of a healthier product, and that there is a risk that women and the broader community will increase their consumption of products that they believe are healthier (Keric *et al*., 2022). This is particularly concerning given the low levels of awareness among the general population of harms associated with alcohol use, including alcohol-caused cancers (Hay *et al*., 2023; Seidenberg *et al*., 2023).

This study also demonstrates that there is a process of symbolic consumption occurring that may influence women’s consumption of ‘low-calorie’ and ‘low-sugar’ alcohol products. There are a range of personal, social and situational factors that women perceive to influence their alcohol consumption. These include drinking to relax and cope with everyday stressors; to build or maintain social connections; or as part of everyday activities such as cooking dinner or enjoying a meal. This is largely consistent with other research that shows that alcohol is seen as an important mechanism for women to build and strengthen social connection and group belonging (Kersey *et al*., 2022; Nicholls, 2022; Wright *et al.*, 2022), and as a way of relieving stress or coping with everyday pressures (McCaul *et al.,* 2019; Ward *et al*., 2022). Alcohol consumption is an important part of individual and group identity for women, with a range of valued, symbolic and ritualistic activities that are associated with and normalize drinking for women. However, the consumption of alcohol is also inconsistent with women’s health goals—particularly related to healthy weight. To combat this, women in this study used ‘low-calorie’ and ‘low-sugar’ alcohol products to reduce their perceived health risks and maintain the social expectation to drink. This study adds to existing literature by showing that the product attributes and promotions associated with ‘better-for-you’ alcohol products resonate with women’s existing health and social values, but also enable them to continue to use alcohol for social connection. In doing so, ‘better-for-you’ products may reinforce dominant social norms that imply that alcohol consumption is important for group belonging and identity (Nicholls, 2022; Wright *et al*., 2022). Women perceived that ‘low-calorie’ and ‘low-sugar’ alcohol products enabled women to participate in alcohol-related events and provided them with a product that they could consume when they were avoiding certain ingredients, on a diet, or being considerate of their weight or calorie intake. Future qualitative research should explore the process of symbolic consumption and ‘better-for-you’ products in more detail and with different subgroups of women.

Australian and New Zealand governments have recognized that some nutrition content and health-oriented claims are problematic in relation to alcohol products. For example, Food Ministers raised concern in 2017 that ‘*sugar claims on alcoholic beverages are misleading and that alcohol is being promoted as a healthier choice for consumers when public health advice is to limit alcohol intake*’ (Food Standards Australia New Zealand, 2018). As a result, Food Standards Australia and New Zealand have begun investigating carbohydrate and sugar claims on alcohol products (Food Standards Australia New Zealand, 2023). Removing such claims will be important in reducing opportunities for consumers to be misled about the health value of alcoholic products. Warning labels on alcohol products could be used to inform consumers of the risks associated with alcohol content (Al-Hamdani and Smith, 2017; Zhao *et al.,* 2020) and could be part of a comprehensive approach to help counter health-related marketing such as nutrition content and health claims. Current warning labels in Australia and New Zealand are limited to communicating the risks of alcohol use during pregnancy. In other countries alcohol warning messages relating to health have included ‘*excess alcohol is damaging to your health*’, ‘*alcohol increases your risk to personal injuries*’ and ‘*drink driving may cause disability or death*’ (AER Foundation, 2011), and Ireland is set to become the first country in the European Union to introduce mandatory health warning labels on all alcohol products (set to come into effect in 2026) (WHO, 2023). Comprehensive public education approaches, which are sustained, free from industry influence and evidence-based, are warranted in protecting women from the harms of the alcohol industry. This approach also includes mandated health warning labels with rotating health messages relevant to women, which could assist in empowering women with accurate knowledge about the risks associated with alcohol products.

### Limitations

The analysis did not look to compare in detail women’s attitudes by age, alcohol intake, alcohol preferences or sociodemographic characteristics. Second, this study only looked at two attributes associated with ‘better-for-you’ products. There is a need to understand the impact and influence of other attributes such as low and zero alcohol options as well as claims such as natural, organic and other health-orientated claims.

## CONCLUSIONS

This study demonstrates that there is a process of symbolic consumption occurring between products that are marketed as ‘low-calorie’ and ‘low-sugar’ and women’s values particularly related to weight and health. This study adds findings to existing literature that shows that ‘better-for-you’ products may continue to normalize the consumption of alcohol by providing products that are viewed as healthier, which may increase women’s consumption of alcohol due to their perception that these products are lower in risk. Regulatory controls should be implemented to remove opportunities for health and nutrition content claims on alcohol products, which may be misleading.

## Supplementary Material

daad184_suppl_Supplementary_File_One

daad184_suppl_Supplementary_File_Two

daad184_suppl_Supplementary_File_Three
