# Supplementary material for: The symbolic consumption processes associated with ‘low-calorie’ and ‘low-sugar’ alcohol products and Australian women
Source: Health Promot Int. 2023 Dec 30;38(6):daad184. doi: 10.1093/heapro/daad184 (PMC10757065; doi:10.1093/heapro/daad184)
Supplement: daad184_suppl_Supplementary_File_One [file daad184_suppl_supplementary_file_one.docx]

**Supplementary File One**

**Application of the Six Steps of Reflexive Thematic Analysis [Braun & Clarke 2022]**

| **RTA Step** | **Application of each step** |
| --- | --- |
| 1. Familiarising yourself with the dataset | Multiple authors read through the dataset as a whole, and subsequently read through the responses to the questions being analysed for this paper to gain an initial understanding of the data. Brief notes were made by each of the authors about any analytical ideas they had while reading through the data set. The authors then met regularly to discuss interesting parts of the data and to question and challenge any assumptions the research team members may have had about their ideas. |
| 1. Coding | Data were then coded to the research questions, researchers identified segments of the data, predominately associated with specific questions, that were interesting and relevant to the research questions. This included codes such as weight, calories, health, diet, and taste. The broader research team actively participated in the decision-making process regarding the analysis by reviewing any decisions that were made.  As the research team became more familiar with the patterns and deeper meanings within the data, our coding shifted from surface level coding towards capturing latent or underlying meanings such as facilitating social connection and scepticism around the true health benefits. At this stage of the thematic analysis process, we revisited symbolic consumption theory to help us better understand our data. |
| 1. Generating initial themes | Codes were then consolidated to create an initial set of themes. The research team combined related codes that shared a similar main concept to form themes that aligned with our overall analytical narrative relating to the research questions. |
| 1. Developing and reviewing themes | During this step, our focus was on ensuring the coherence and relevance of our themes in relation to our research questions and the overall narrative that the research team were constructing from the data. Each theme was reviewed to ensure the theme told a convincing story about the pattern of shared meaning across participant responses, and that the themes together highlighted the most important patterns across the dataset. |
| 1. Refining, defining and naming themes | To ensure the themes were distinct from one another and had clear boundaries, concise summaries of each potential theme were written, capturing the essence and uniqueness of these themes. At this stage the research team named the themes and developed a model to demonstrate the way the findings were aligned with symbolic consumption theory. |
| 1. Writing up | Finally, the analysis was written up and presented as a narrative to describe the symbolic consumption processes associated with women’s consumption of ‘low calorie’ and ‘low sugar’ alcohol products. The analysis is supported by extracts of the data and discussed in relation to the research questions and existing literature. |
