# Supplementary material for: The symbolic consumption processes associated with ‘low-calorie’ and ‘low-sugar’ alcohol products and Australian women
Source: Health Promot Int. 2023 Dec 30;38(6):daad184. doi: 10.1093/heapro/daad184 (PMC10757065; doi:10.1093/heapro/daad184)
Supplement: daad184_suppl_Supplementary_File_Two [file daad184_suppl_supplementary_file_two.docx]

**Supplementary File Two: Summary of key themes**

| **Theme** | **Sub-theme** | **Illustrative quotes** |
| --- | --- | --- |
| **Reasons for drinking alcohol** | | |
| *A mechanism for relaxing and coping with daily stressors* | Drinking to relax and cope with stress | - To let loose, to wipe my mental state and relax. – 32-year-old - Relax take my mind off things. – 53-year-old - A stress reliever after a long day or if something bad has happened, it takes the pain away. – 20-year-old |
|  | Consumption associated with everyday activities | - I only have an occasional drink while watching sports on tv or movie. – 60-year-old - It finishes off my day before I cook and have dinner.it relaxes me and puts me in a good place. – 63-year-old - I like it and it makes me feel comfortable, something to look forward to at the end of the day. – 76-year-old |
| *Social practices, group connection, and social rituals* | Drinking to facilitate social connection | - To be social with family and friends. – 24-year-old - Social function with friends and work colleagues. – 55-year-old - For fun and to be social. – 45-year-old - To socialise and meet new people. – 27-year-old |
|  | Drinking to get drunk and feel intoxicated | - To either have some fun or because I just felt like getting drunk. – 24-year-old - To get a buzz. – 25-year-old - To get drunk. – 44-year-old |
| *Reasons for reducing alcohol consumption* | Parental responsibilities | - My child, I hate drinking while he’s around, and I'm a full-time mum, so i very rarely drink. – 33-year-old - I didn’t drink while I was pregnant and very rarely while nursing. Now have low tolerance for alcohol and can’t consume too much as I need to be focused for kids. – 74-year-old |
|  | Negative experiences | - I don’t drink a lot cause my father use to drink every day. – 23-year-old - My brother that’s why I don’t drink a lot as he drinks far too much. – 24-year-old |
| **Engagement with and perceptions of ‘low calorie’ and ‘low sugar’ alcohol products.** | | |
| *An alternative for those looking for a ‘healthier’ option* | A healthy alternative and aligned with health-related values. | - Think that they are better for them. –59-year-old - They will want it more because it is somewhat healthier. – 18-year-old - Seems as a ‘healthier’ option. – 36-year-old - Health benefits. – 24-year-old |
|  | Perception women could feel less guilty | - Makes it seem less ‘naughty’ when dieting. – 38-year-old - Women who want to lose weight or maintain their weight may enough this as a guilt free way to drink. – 20-year-old |
|  | Scepticism around the true health benefits | - Oh, let’s drink and be skinny pfft. – 53-year-old - Weight conscious woman would definitely care, they’d be better off not drinking. – 33-year-old - Might lull them into thinking its healthier. – 36-year-old - False pretence that it's better for weight. – 34-year-old |
| *Symbolically aligning with values about body weight and the thin ideal* | Positive impacts on weight loss and weight management | - To assist with weight loss. – 29-year-old - Trying to lose weight but still wanted to be able to have a drink without too many calories. – 22-year-old - To help prevent weight gain. – 62-year-old - Appeal of it being less calories and fitting in with daily calories intake if I was planning on losing weight. – 26-year-old - No one wants to put on weight. – 58-year-old |
|  | Diet culture and alignment with women’s insecurities relating to appearance | - Continues to push the ‘only thin women are beautiful’ agenda. – 24-year-old - It’ll get more women on board as a result of diet culture. – 26-year-old - I think that low calorie/low sugar products would somewhat influence women's alcohol use as despite the indulgence, women like to take care of themselves. – 50-year-old - A lot of young women have body dysmorphia issues and could be tempted by these drinks by still being able to drink without the fear of putting weight on. – 52-year-old |
| *Normalising drinking through increased social acceptance and a reduced perception of risk* | Increasing consumption due to perceptions of lower calories and sugar | - Makes them drink more without worrying about calories. – 60-year-old - Encourage more drinking as they don’t feel they’re putting on empty calories. – 23-year-old - It suggests that drinking low/no sugar calorie alcohol is better therefore can be consumed in greater amounts. – 52-year-old - Increase uptake. If I know one drink only has 80 calories, then I know I can consume more of them in comparison to say one cocktail that might have 400 calories. – 26-year-old |
|  | Encouraging alcohol use | - More women may be enticed to drink. – 25-year-old. - A lot...then i might drink twice as much. – 48-year-old - They may think they can drink more. – 57-year-old - They may drink more than they should. – 33-year-old - Possibly can influence women to buy more of 1 and get more drunk, not having to worry about calories. – 19 year old |
