# Supplementary figures and images for: The symbolic consumption processes associated with ‘low-calorie’ and ‘low-sugar’ alcohol products and Australian women
Source: Health Promot Int. 2023 Dec 30;38(6):daad184. doi: 10.1093/heapro/daad184 (PMC10757065; doi:10.1093/heapro/daad184)

Supplementary File Three: Model


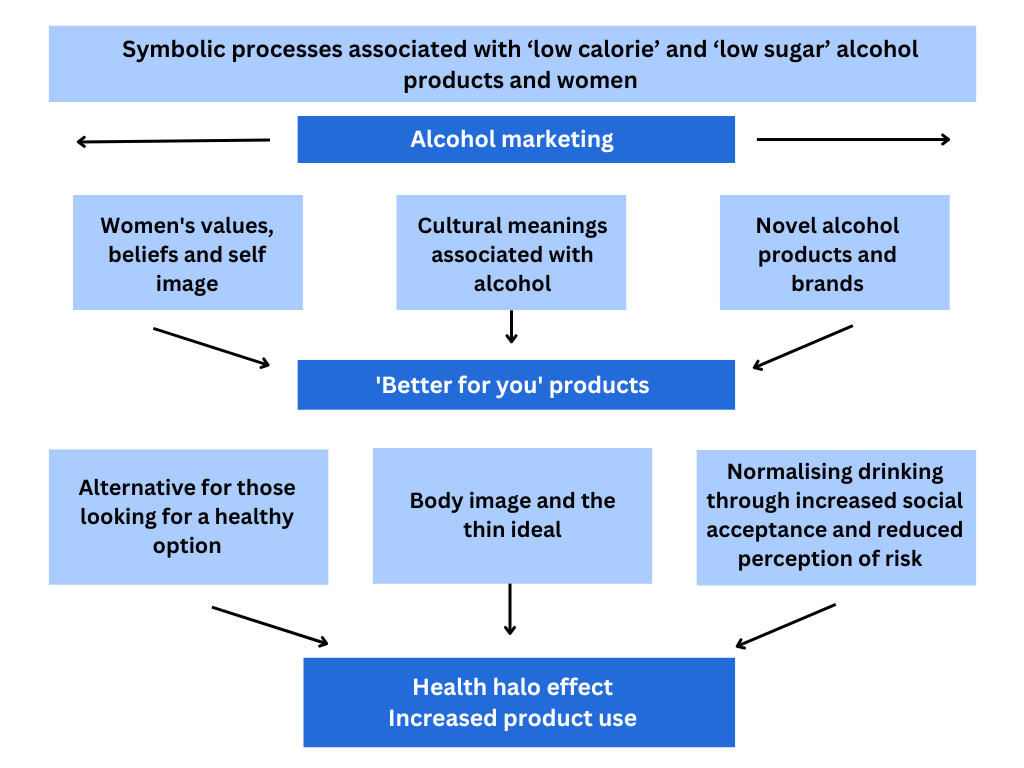

Supplement: daad184_suppl_Supplementary_File_Three [file daad184_suppl_supplementary_file_three.docx]
